# Editorial: Advancement in Gene Set Analysis: Gaining Insight From High-Throughput Data

**DOI:** 10.3389/fgene.2022.928724

**Published:** 2022-05-26

**Authors:** Farhad Maleki, Sorin Draghici, Renee Menezes, Anthony Kusalik

**Affiliations:** ^1^ Augmented Intelligence & Precision Health Laboratory, Department of Radiology and Research Institute of the McGill University Health Centre, Montreal, QC, Canada; ^2^ Department of Computer Science, Wayne State University, Detroit, MI, United States; ^3^ Biostatistics Centre and Department of Psychosocial Research and Epidemiology, Netherlands Cancer Institute, Amsterdam, Netherlands; ^4^ Department of Computer Science, University of Saskatchewan, Saskatoon, SK, Canada

**Keywords:** gene set analysis, pathway analysis, enrichment analysis, gene expression, next-generation sequencing

The existence of high-throughput technologies allows for the study of a large number of genes in a single experiment. However, analyzing such high-throughput data and interpreting the results are challenging (Draghici, 2016).

Phenotypes or biological conditions often result from the coordinated activity of a group of genes or biomolecules. Consequently, the study of the coordinated expression pattern of biologically related genes is essential for understanding the mechanisms underlying these conditions or phenotypes. Knowledge bases such as GO (Consortium, 2004) and KEGG (Kanehisa and Goto, 2000) aim to capture knowledge about the roles that genes play in various biological processes and locations. Such resources can be generally divided into: 1) gene set databases (e.g., GO), which include only associations between genes and annotations such as biological processes; and 2) pathway databases (e.g., KEGG), which also capture knowledge related to the interactions between the genes.

Various categories of methods have been developed over time to extract knowledge from such resources (Maleki et al., 2020). The very first methods used a simple approach to identify the gene sets that are enriched in differentially expressed genes (Khatri et al., 2002; Dennis et al., 2003; Draghici et al., 2003b). This approach has various limitations including the fact that it ignores the magnitude of the measured gene expressions. This was addressed by the second generation of methods, pioneered by GSEA (Subramanian et al., 2005), and called functional class scoring (FCS). FCS methods use the correlation between gene expression and the phenotype but still ignore all the interactions between genes. This was addressed by the third generation of methods, called topology-based, or pathway analysis methods. The first such method, impact analysis (Draghici et al., 2007; Tarca et al., 2009), was soon followed by a plethora of over 20 other approaches (Khatri et al., 2012; Mitrea et al., 2013; Nguyen et al., 2018). Many of these methods have been bench-marked recently (Nguyen et al., 2019).

Even though pathway analysis methods are very different from enrichment and FCS methods, we will use “gene set analysis” to generically refer to the entire family of methods aimed at understanding the coordinated expression pattern of known gene sets or pathways. Despite the widespread use of gene set analysis, little consensus exists in the research community regarding best practices. This Research Topic is aimed at highlighting methodological advances as well as applications of gene set analysis to improve the utility of these methods in gaining insight from high-throughput expression studies. Highlights are as follows.

Testing for case-control gene expression differences between two groups is a common approach in studies in which researchers are interested in the “difference of differences”. Weiner et al. describe a frequent methodological error in using and interpreting gene set analysis methods for such studies. The error occurs when researchers test for differential expression separately in each group and consider genes with significant expression differences in only one comparison—i.e., one group—specific to that group. Based on this assumption, a gene set enrichment analysis is used to find gene sets/pathways specific to only one group. Weiner et al. empirically show that such an approach could report differentially enriched gene sets even for scenarios with no statistically significant differences between the groups.

Marczyk et al. evaluate the effect of incorporating different approaches for integrating single-nucleotide polymorphism (SNP) information and linkage disequilibrium correction on the performance of several gene set analysis methods. They suggest that linkage disequilibrium correction and Stouffer integration could improve the performance of gene set analysis for genome-wide association studies.

Several articles focus on gene set analysis for cancer research. Luo et al. use GSEA (Subramanian et al., 2005) to study the pathways associated with DNA methylation-derived differentially expressed genes in patients with prostate cancer. Song et al. also identify a ubiquitin-related gene signature for prostate cancer prognosis. Li et al. study the association of S100 genes with well-known tumor-related pathways. Xu et al. utilize gene set analysis to identify biological functions and pathways associated with the ferroptosis-related genes in patients with skin cutaneous melanoma. Tan et al. use GSEA to identify gene sets associated with genes co-expressed with the SBSN gene. He et al. find genes differentially expressed in patients with renal cell carcinoma to be associated with autophagy-related pathways. They suggest a prognosis risk score for renal cell carcinoma based on autophagy-related genes that are differentially expressed in patients with the cancer.

The applications of gene set analysis are not limited to cancer research. Yousef et al. employ gene set analysis to validate the biological relevance of the results of their algorithm for miRNA-mRNA regulatory module detection. Du et al. identify hub genes and pathways implicated in osteoporosis. Wu et al. explore potential hub genes in non-alcoholic fatty liver disease and gene sets associated with these genes.

Due to the complex nature of gene set analysis, developing tools that conduct gene set analysis and facilitate interpreting its results is valuable. Among tools commonly used for gene set analysis are DAVID (Dennis et al., 2003), Enrichr (Kuleshov et al., 2016), WebGestalt (Liao et al., 2019), iPathwayGuide (Ahsan and Draghici, 2017), and Onto-Tools (Draghici et al., 2003a). In this Research Topic, Yue et al. present “PAGER Web APP” as an interactive web-based application supporting online R scripting of integrative gene set analysis, and Odom et al. develop an R Package for integrative analysis of multi-omics datasets offering the functionality to work with matched or non-matched samples.

Despite the existence of a large number of gene set analysis methods, there is little consistency among different methods when analyzing the same gene expression dataset (Maleki et al., 2019b; Nguyen et al., 2019). Although gene set overlap is a common phenomenon in gene set databases, most gene set analysis methods disregard such an overlap. This results in a lack of specificity of these methods (Maleki et al., 2020).

Evaluating gene set analysis methods is extremely important (Zyla et al., 2016, 2019) However, most gene set analysis methods have been evaluated either based on oversimplified data—which do not represent real expression datasets and real gene set knowledge bases—or based on real expression datasets with presumed enrichment status for gene sets. Maleki et al. (2021) developed Silver as a methodology for evaluating such methods without relying on oversimplifying assumptions. Besides a thorough evaluation, new gene set analysis methods need to be systematically assessed to find the minimum number of samples required to achieve reproducible results (Maleki et al., 2019a).

The papers published in this Research Topic indicate that the development of gene set analysis methods and tools remains an active research area.
